# MethParquet: an R package for rapid and efficient DNA methylation association analysis adopting Apache Parquet

**DOI:** 10.1093/bioinformatics/btae410

**Published:** 2024-06-19

**Authors:** Ziqing Wang, Michael Cassidy, Danielle A Wallace, Tamar Sofer

**Affiliations:** Department of Medicine, Cardiovascular Institute, Beth Israel Deaconess Medical Center, Boston, MA, 02215, United States; Department of Medicine, Cardiovascular Institute, Beth Israel Deaconess Medical Center, Boston, MA, 02215, United States; Department of Medicine, Cardiovascular Institute, Beth Israel Deaconess Medical Center, Boston, MA, 02215, United States; Division of Sleep Medicine, Harvard Medical School, Boston, MA, 02115, United States; Division of Sleep and Circadian Disorders, Department of Medicine, Brigham and Women’s Hospital, Boston, MA, 02115, United States; Division of Sleep and Circadian Disorders, Department of Neurology, Brigham and Women’s Hospital, Boston, MA, 02115, United States; Department of Medicine, Cardiovascular Institute, Beth Israel Deaconess Medical Center, Boston, MA, 02215, United States; Division of Sleep Medicine, Harvard Medical School, Boston, MA, 02115, United States; Division of Sleep and Circadian Disorders, Department of Medicine, Brigham and Women’s Hospital, Boston, MA, 02115, United States; Division of Sleep and Circadian Disorders, Department of Neurology, Brigham and Women’s Hospital, Boston, MA, 02115, United States; Department of Biostatistics, Harvard T.H. Chan School of Public Health, Boston, MA, 02115, United States

## Abstract

**Summary:**

Genome-wide DNA methylation (DNAm) profiling is indispensable for unveiling how DNAm regulates biological pathways and individual phenotypes. However, managing and analyzing extensive DNAm data generated from large cohort studies present computational obstacles. Apache Parquet is a data file format that allows for efficient data storage, retrieval, and manipulation, alleviating computational hurdles associated with conventional row-based formats. We here introduce *MethParquet*, the first R package leveraging the columnar Parquet format for efficient DNAm data analysis. It can be used for data extraction, methylation risk score calculation, epigenome-wide association analyses, and other standard post-quality control tasks. The package flexibly implements diverse regression models. Via a public methylation dataset, we show the efficiency of this package in reducing running time and RAM usage in large-scale EWAS.

**Availability and implementation:**

The MethParquet R package is publicly available on the GitHub repository https://github.com/ZWangTen/MethParquet. It includes a vignette and a toy dataset derived from a public resource.

## 1 Introduction

DNA methylation (DNAm) is essential for various fundamental biological processes including transcriptional regulation, embryonic development, genomic imprinting, and chromosomal inactivation (Yong, Hsu and Chen 2016). In humans, up to 80% of the cytosine-phosphate-guanine dinucleotides (CpGs) are methylated, predominantly as 5-methylcytosine, which results from adding a methyl group to the fifth carbon of cytosine (Yong, Hsu and Chen 2016). Moreover, these epigenetic marks are dynamic and reprogrammable throughout the life cycle and can be transgenerational (Hackett and Azim Surani 2013). Genome-wide DNAm profiling is performed to explore the associations between methylated genomic regions and metabolic phenotypes and diseases. However, processing and analyzing large methylation datasets poses computational challenges.

**Figure 1.**
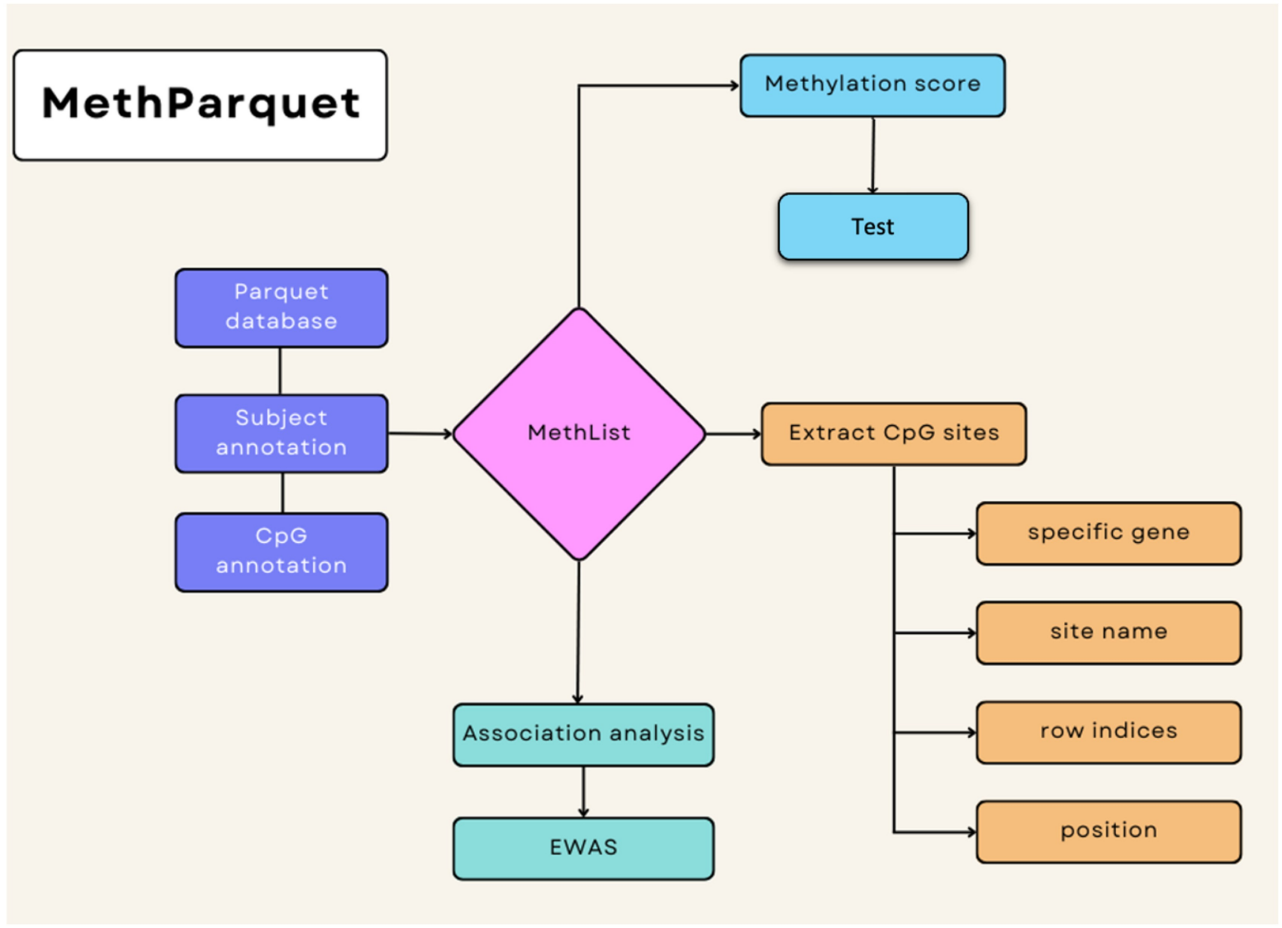
Functionality schema of *MethParquet* package. MethList consists of the open connection to methylation Parquet database, subject, and CpG annotation objects. Then it can be utilized as input to calculate methylation risk score, perform association analysis, or extract a subset of methylation data given different criteria.

Apache Parquet is an open-source, columnar file format created for efficient data storage and usage. Unlike row-oriented formats, Parquet stores and compresses data column-by-column, optimizing query performance, and input/output by minimizing loaded data. This accommodates fast data manipulation and retrieval. Parquet also compresses data efficiently, reducing both temporary and permanent memory needs. In addition, it is free to use and compatible with numerous R functions including *dplyr* (Wickham *et al.* 2023) (https://github.com/tidyverse/dplyr) to facilitate data manipulation. Accordingly, we propose storing and analyzing preprocessed DNAm data, that has already been checked for quality and normalized, in Parquet format. We have developed an R (R Core Team 2023) (https://www.R-project.org) package, *MethParquet*, which implements an object called Methlist to expedite analyses. This Methlist object connects a generated Parquet database with CpG and subject-level annotations. It can then be used to perform epigenome-wide association analysis (EWAS), calculate methylation risk scores (MRS), and extract CpG site subsets (Fig. 1). By avoiding loading all data at once, *MethParquet* enables faster handling and analysis of large methylation datasets.

## 2 Materials and methods

To demonstrate the usage of *MethParquet* we use publicly available DNAm data, along with body mass index (BMI) measures from the EWAS Data Hub (Xiong *et al.* 2020). The BMI methylation data contains over 420 000 CpG sites across 11 different tissues from 2070 subjects. Sample and CpG annotations were obtained from EWAS Data Hub (Xiong *et al.* 2020) and Illumina respectively. As the full dataset is utilized in the analysis for the purposes of timing and memory comparison, rather than to reveal relationships of CpG methylation with BMI, association results will not be described. A running example of using the MethParquet package is provided in the Supplementary Information.

### 2.1 MethList creation and extraction of methylation data

*MethParquet* operates under the assumption that the DNA methylation (DNAm) data are pre-processed and quality-checked, as it does not provide functionality for data normalization. The function *write_parquet_meth* is created to facilitate the construction of Parquet database without loading the methylation data into local memory, leveraging the *write_dataset* function from the *arrow* package v13.0.0 (Richardson *et al.* 2023) (https://github.com/apache/arrow/). It's important to note that the *MethParquet* workflow mandates that the methylation data contain a column specifying the chromosome number to ensure compatibility with downstream analyses. To use the methylation Parquet database, we open a connection to it via *open_dataset* function in *arrow*. This open connection becomes an element in the MethList object. The MethList also includes two annotation objects at the sample- and CpG-level, incorporated using the create_meth_list function. Users can choose to include relevant annotation file columns. When working with big data in cloud storage, the *create_methlist_S3* function is designed so that users can link and access their Parquet database stored in Amazon Web Services (AWS) Simple Storage Service (S3) without the overhead of downloading large datasets onto their local machines. This function is enabled by utilizing the *aws.s3* v0.3.21 (Leeper 2020) and *arrow* v13.0.0 (Richardson *et al.* 2023) *packages.*

Relying on the CpG annotation, *MethParquet* enables CpG site extraction by gene or CpG name, chromosome number, and row indices. The extracted methylation data and CpG annotations are returned in “data frame” format.

### 2.2 Methylation risk score

Calculation of a phenotype-specific methylation risk score (MRS) is implemented in *dev_meth_score*. The MRS is defined as a weighted sum of an individual’s DNAm values and pre-calculated weights associated with the phenotype of interest (Hüls and Czamara 2020). Options to scale the MRS or divide it by the number of methylation sites are also available. The constructed MRS can then be tested as an exposure for its relationship with another phenotype, whether continuous or categorical, by fitting a linear, logistic or multinomial regression model, respectively. These regression models were made available with *stats* (R Core Team 2023) and *nnet* v7.3.19 (Venables *et al.* 2002) (https://www.stats.ox.ac.uk/pub/MASS4/) packages. A likelihood ratio test was implemented for multinomial regression model using the *ANOVA* function in the *car* package v3.1.2 (Fox and Weisberg 2019) (https://socialsciences.mcmaster.ca/jfox/Books/Companion/). Users can also specify covariates to adjust for when fitting the models. The computed MRS can also be used in any downstream analyses that does not rely on the *MethParquet* package. For demonstration purposes, we calculated MRS for C-reactive protein (CRP) using pre-developed weights of CpG sites associated with blood CRP level (Hillary *et al.* 2023). We then tested the association between this MRS and BMI.

### 2.3 Association analysis

When treating methylation values as outcomes, association analysis can be conducted on either the full set or a subset of DNAm data using *lm_ewas_outcome* or *rlm_ewas_outcome*, using linear regression or robust linear regression to account for potential outliers. Robust M-estimation is enabled through the *limma* package v3.56.2 (Ritchie *et al.* 2015). Continuous phenotypes undergo a t-test while categorical outcomes associations are tested via an F-test. Multiple testing correction for each CpG is carried out with the Benjamini-Hochberg (BH) method (Benjamini and Hochberg 1995). These EWAS functions also allow for covariate adjustment to control for confounding of genetic and non-genetic factors such as cell-type composition, age, sex, etc.

Users can also apply the *ewas_meth_exposure* function to test associations while the methylation values are treated as exposures. This involves first fitting null models via simple linear, generalized linear, or mixed linear regression with the *NullModel* function. The latter two were implemented from the existing *stats* (R Core Team 2023) and *GENESIS* v2.30.0 (Gogarten *et al.* 2019) packages. The mixed linear model allows to incorporate random effects like pairwise individual relatedness. Strengths of CpG associations are then evaluated by score tests, adapted from the *GENESIS* (Gogarten *et al.* 2019) and *statmod* package v1.5.0 (Giner and Smyth 2016), followed by multiple testing correction by using the Benjamini-Hochberg false discovery rate controlling procedure (Benjamini and Hochberg 1995).

To mitigate computational burden, the Parquet database is queried in user-customizable or default 50 000-CpG blocks for EWAS functions. Moreover, MethParquet efficiently solves the linear regression with ordinary least square method through matrix and algebraic operations implemented in C++ code via *Rcpp* v1.0.12 package (Eddelbuettel and François 2011). This approach accelerates the *lm_ewas_outcome* function by testing CpG sites in bulk and without the need to compute additional parameters. In contrast, robust linear regression performs the association test site by site.

For users operating on Linux or macOS systems, they also have the option to set number of cores used for parallel processing rendered by *furrr* v0.3.1 (Vaughan and Dancho 2022) (https://github.com/DavisVaughan/furrr 2022) and *future* v1.33.1 (Bengtsson 2021) packages, which further reduces runtime and memory usage. Users familiar with future strategy can leave the argument “parallel” to its default value of 1 for sequential processing, so their future backends remain unaffected.

In addition, we provide a flexible implementation of association analysis through *flex_ewas*, which accepts a regression function as input to carry out a “site by site” association test. Therefore, the users have the autonomy and flexibility to apply any external models/tests, despite a trade-off in computational efficiency.

### 2.4 Comparison with available packages

To compare computational performance between packages relying on Parquet and row-based data format, we conducted EWAS using *MethParquet* in RStudio (v2023.6.2.561) and GLINT v1.0.4 (Rahmani *et al.* 2017) in Python v2.7 (Van Rossum and Drake Jr 1995) on a personal computer MacBook Pro with a 5 GHz processor and 8 GB of RAM. Specifically, a linear model was fitted with BMI as the exposure and methylation as the outcome without adjusting for covariates. We repeated the experiments 10 times and monitored the time elapsed and RAM usage for the entire process, from data loading to EWAS, using the *peakRAM* package (Quinn 2017) (http://github.com/tpq/peakRAM). Given that processing all the data in row-based format was impractical on a personal computer at this scale, same comparative analysis was also conducted against two widely used packages, *Minfi* v1.46.0 (Aryee *et al.* 2014) and *limma* v3.56.2 (Ritchie *et al.* 2015) on a single AWS instance r5a.2xlarge with 2.5 GHZ sustained clock speed and 64 GB of RAM.

## 3 Results and discussion

### 3.1 Methylation risk score construction and testing

MRS construction was conducted 10 times on the same personal computer to obtain the average runtime and RAM usage (Supplementary Table S1). Remarkably, computation of MRS involving 1468 CpGs associated with CRP took <1 min with a peak RAM of 6 MiB. The utilization of Parquet data hereby offers a significant advantage by circumventing the need to load the entire methylation matrix for the calculation. Instead, only relevant CpGs were queried from the database. Function *test_mrs* was then used to examine the association between the scaled CRP MRS and BMI via linear regression, yielding a coefficient of 1.6 kg/m^2^ per 1 standard deviation increase in the CRP MRS (t = 7, *P* < 0.001).

### 3.2 Association analysis performance

The entire EWAS pipeline was assessed, spanning data loading through significance testing. The analysis of *MethParquet* was completed in RStudio without parallel processing due to stability concerns within the RStudio environment (Bengtsson 2021). With a block size of 50 000, the MethParquet EWAS on the personal computer was completed in 12 min, with a peak RAM usage of 1783 MiB (Table 1). Although *MethParquet* is slower than the python-based package GLINT (Table 1), it provides users with lower peak RAM and with higher flexibility in performing EWAS on subsets of CpGs, which can be treated as either outcome or exposure. While EWAS using GLINT only models CpG as exposure, thus limiting the biological interpretation from the direction of the association.

**Table 1.** Benchmark results of the cascade oscillators model.^a^

	Time (s)	Peak RAM (MiB)
**AWS (mean, median)**		
MethParquet	484.2, 484.5	220.7, 224.5
Minfi	669.5, 664.7	22118.0, 21804.8
Limma	801.8, 796.3	37782.8, 38496.4
**Personal computer (mean, median)**		
MethParquet	739.3, 739	1782.8, 1783.5
GLINT	507.7, 500	3276.7, 3205.1

a RAM: random-access memory. s, seconds; MiB, mebibytes*.*

On AWS, *MethParquet* running on four parallel cores outperformed *Minfi* and *limma* in both runtime and RAM usage (Table 1). In terms of EWAS results, all packages yielded identical coefficient estimates (Supplementary Fig. S1). The most prominent advantage of *MethParquet* lies in its efficient RAM usage, as shown in Table 1. Furthermore, EWAS via *MethParquet* is expected to be even faster when working with locally stored data. We observed a runtime decrease of 3 min executing *lm_ewas_outcome* on the AWS head node where the Parquet database is stored. That said, the same analysis connecting Parquet data stored in AWS S3 bucket took around 50 mins to finish on the personal computer.

The most time- and memory-consuming step in an EWAS is the data query and processing. *MethParquet* addresses this challenge by (i**)** using the Parquet format for storing and loading data, (ii**)** efficiently implementing regression tests, and (iii**)** partitioning the analysis to “chunks,” as is often done in genome-wide association studies. Consequently, only a portion of the data was loaded at a time. Another factor contributing to the disparity in memory usage is likely attributed to data compression. The use of Parquet resulted in a 1:2 compression gain compared to the original 6-gigabyte (GB) text file. In other words, packages built on row-based data would acquire additional RAM to complete the analysis as *MethParquet.* That said, the efficiencies gained through data partitioning and compression could ultimately lead to substantial cost savings, especially in the context of cloud computing services.

In case of potential outliers and samples with extreme methylation levels, users can apply robust linear regression using the *rlm_ewas_outcome* function, which is more robust in the presence of outlier observations. As *rlm_ewas_outcome* only tests one CpG site at a time, it is slower. Users can also fit mixed linear model through the *NullModel and ewas_meth_exposure* functions to account for one or multiple random effects that induce correlations between the samples.

Another function, *flex_ewas* offers users greater flexibility in incorporating external functions to conduct EWAS on their Parquet data. For instance, robust linear regression (*rlm*) from the *MASS* package was applied in the running example instead of our own *rlm_ewas_outcome* function (see Supplementary Information). All EWAS results are presented in a consistent manner, listing all tested CpG sites, effect-size estimates, test scores, *P*-values and BH corrected values (see Supplementary Information).

Other available software capable of conducting EWAS exist. For example, factored spectrally transformed linear mixed models (FaST-LMM) has its runtime and memory use scaled linearly with cohort size rather than number of sites (Lippert *et al.* 2011), which is also implemented in GLINT (Rahmani *et al.* 2017). Moreover, its EWAS version FaST-LMM-EWASher was created to automatically correct for cell-type composition (Zou *et al.* 2014). In addition to single-trait EWAS, methylSCOPA utilizes reverse regression approach to study associations between DNAm with multiple correlated phenotypes (Draisma *et al.* 2019). These two packages were not included in the performance assessment due to their specific requirements in data format and operating system (Lippert *et al.* 2011, Zou *et al.* 2014, Draisma *et al.* 2019), which differ from those utilized in the current study. However, we recognize the importance of these software and encourage further exploration of their capabilities in appropriate contexts.

To the best of our knowledge, *MethParquet* is the first R package to encompass a diverse range of DNA methylation analyses in Parquet format. It features MRS calculation and testing as well as EWAS to assist researchers in conducting association studies. To maintain a seamless analysis pipeline, the output from EWAS can be readily used for visualization such as QQ and Manhattan plots, based on user-specified columns.

In conclusion, *MethParquet*, coupled with a Parquet database of methylation data, serves as a swifter and more efficient alternative to standard EWAS approaches in R that rely on a single data object containing the entire methylation dataset. After initial preparation and QC of DNAm data, they can be converted to a Parquet file format for more efficient downstream analysis with MethParquet. *MethParquet* harnesses the advantages of the columnar Parquet format, as opposed to the row-based data format utilized by other existing packages. This package offers great flexibility in analyzing DNAm, from linear regression and mixed model for association analysis, to DNAm scores construction. In summary, MethParquet may be a useful tool for DNAm analysis, especially for analyses of larger datasets where computational efficiency and cost can be a limiting factor.

## Supplementary Material

btae410_Supplementary_Data

## Data Availability

The datasets underlying this article are available in [EWAS Data Hub] at https://ngdc.cncb.ac.cn/ewas/datahub.
