# Supplementary material for: MethParquet: an R package for rapid and efficient DNA methylation association analysis adopting Apache Parquet
Source: Bioinformatics. 2024 Jun 19;40(7):btae410. doi: 10.1093/bioinformatics/btae410 (PMC11219476; doi:10.1093/bioinformatics/btae410)
Supplement: btae410_Supplementary_Data [file btae410_supplementary_data.pdf]

MethParquet: An R Package for Rapid and Efficient DNA Methylation Association Analysis Adopting Apache Parquet:
Supplementary Information

Supplementary Figure 1
Supplementary Table 3
A running example 3
Association Analysis (EWAS) 3
Simple and robust linear regression 4
Mixed linear model 6
Flexible EWAS 8
Using MethParquet for Methylation Risk Score and Differentially Methylated Regions 8
Methylation Risk score (MRS) 9
Differentially Methylated Regions (DMRs) 10

Supplementary Figure

A)

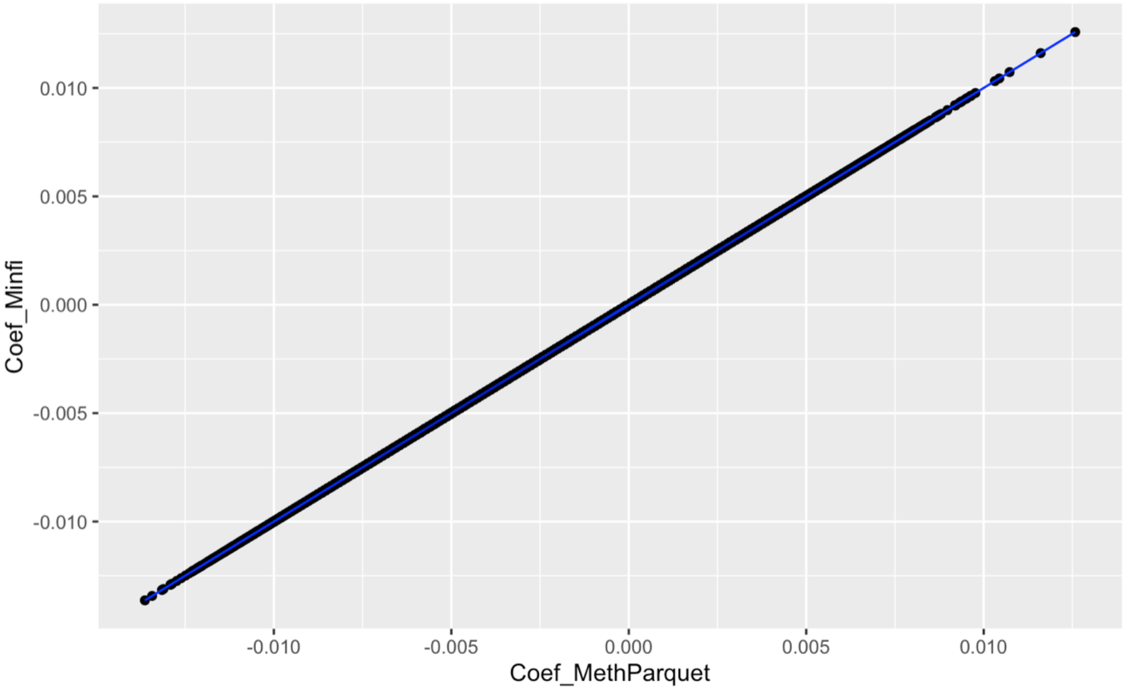

B)

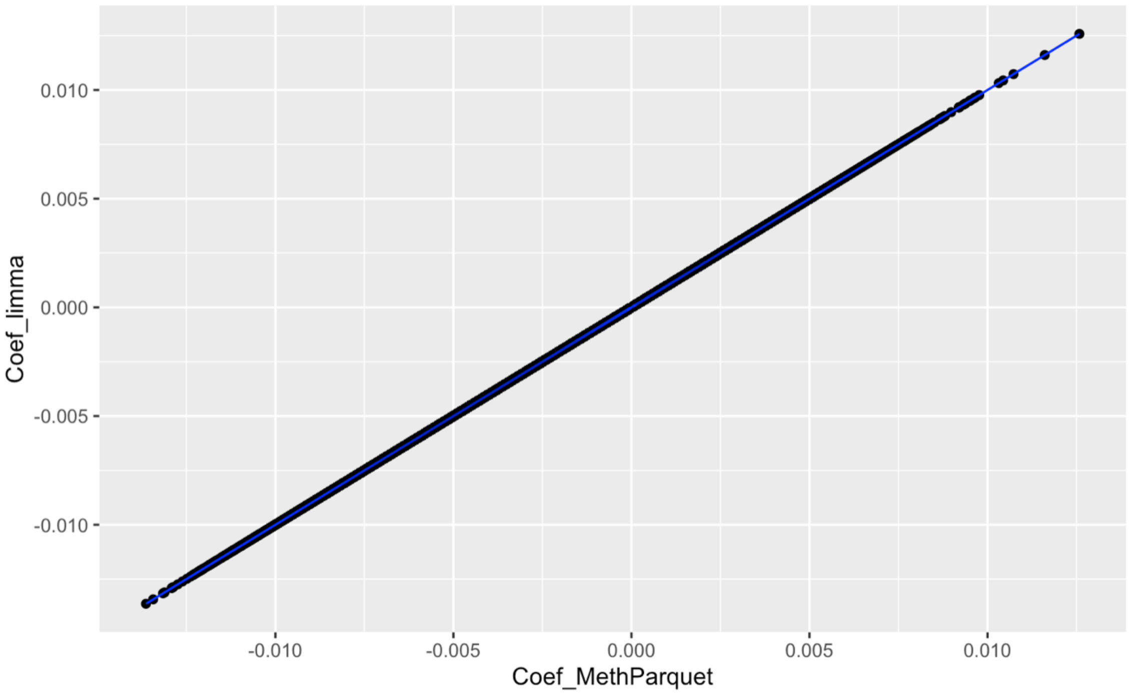

0.010
0.005
0.000
-0.005
-0.010
Coef_limma
-0.010
-0.005
0.000
0.005
0.010
Coef_MethParquet

C)

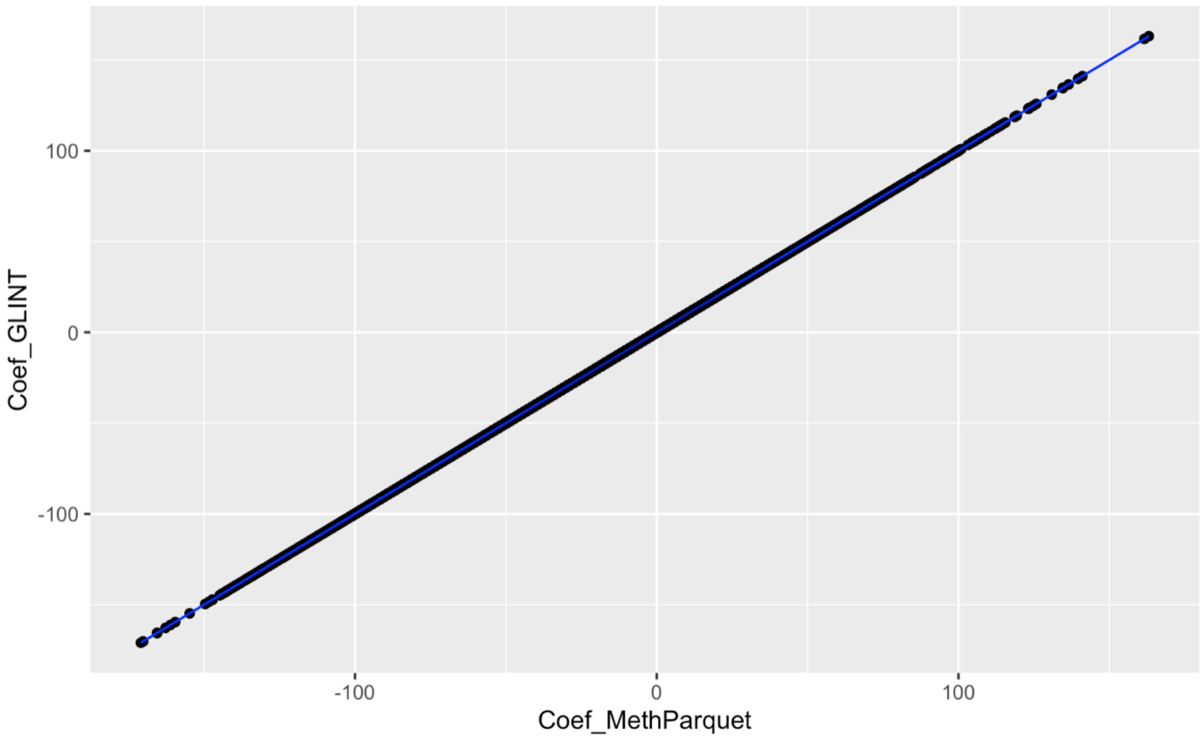

100
0
-100
Coef_GLINT
-100
0
100
Coef_MethParquet

Figure S1. Coefficient estimates obtained using *dmpFinder* of *Minfi* (A), *lmFit* of *limma* (B), *GLINT* (C) and *lm_ewas_outcome* of *MethParquet,* fitting the same model DNAm ~ BMI. Comparison between GLINT and MethParquet was based on EWAS results with CpG as exposure (C).

Supplementary Table

Table S1. Runtime and random-access memory for methylation risk score construction using *MethParquet*

	**Time (s)**	**Peak RAM (MiB)**
Mean	43.9	6.2
Median	42.1	6.2

A running example

Association Analysis (EWAS)

The first part of the running example shows how to use MethParquet to conduct association tests on normalized methylation data, from simple linear to mixed models incorporating kinship matrix as random effect to account for genetic similarity among samples, inspired by GENESIS. We also provide a short example for a more flexible association analysis using `flex_ewas()`, which accepts a regression model created by users as input to test each CpG site one after another.

Load required packages

```
library(MethParquet)
library(arrow)
library(tidyverse)
library(limma)
library(GENESIS)
library(knitr)
```

Create Parquet database and MethList object

MethParquet takes a `MethList` object created by `create_methlist()`. This object must contain the connection to the Parquet database for the processed methylation data (in beta or M values), along with sample and CpG annotation in the `data.frame` format. Below is the example code to create the parquet database using `write_parquet_meth`, leveraging the package arrow, followed by generation of MethList. Note that MethData is only loaded to demonstrate the data structure. During creation of the Parquet database, this methylation matrix is not loaded into local memory.

```
# Load example data
data(phenoData)
```

```
data(chrAnnotation)
MethData <- read.csv(system.file("extdata/MethData.csv", package="MethParquet"))
kable(head(MethData,c(3L,5L)))
```

CpG	CHR	GSM2350716	GSM1504930	GSM1174899
cg09441152	18	0.017	0.035	0.023
cg11023721	18	0.016	0.032	0.024
cg02935272	18	0.952	0.869	0.952

Create Parquet database in `path` where the methylation data is partitioned by chromosome.

```
wdir <- getwd()
methpath <- paste0(wdir,'/inst/extdata/MethData.csv')
path <- paste0(wdir,'/Parquet_Directory')
write_parquet_meth(data_path=methpath,format='csv',group_by='CHR',parquet_path = path)
```

While creating MethList, users can choose to include only relevant columns from CpG and sample annotation to optimize data. Here only CpG names and position in chromosome (`Name` and `MAPINFO`), choromosome number `CHR` as well as gene names `UCSC_RefGene_Name` are included in the `mlist`.

```
names(chrAnnotation)[c(2,12:13,16)]
#> [1] "Name"              "CHR"               "MAPINFO"
#> [4] "UCSC_RefGene_Name"
mlist <- create_methlist(db_path = path,cpg_col_db='CpG',subject_annot = phenoData,
subject_col_keep='all',cpgAnnot_col_keep=c(2,12:13,16),cpg_annot = chrAnnotation,
subject_id='sample_id',cpg_col_annot='Name', gene_col_name = 'UCSC_RefGene_Name')
names(mlist)
#> [1] "db"            "subject_annot" "cpg_annot"
```

Association analysis

MethParquet provides various models and ways to perform association analysis on methylation data. In this vignette we will show both the built-in models, as well as the flexible implementation using external ones. To streamline the pipeline, output of these analysis can be readily visualized by constructing QQ or Manhattan plots.

Simple and robust linear regression

`lm_ewas_outcome()` and `rlm_ewas_outcome()` regresses methylation on phenotypic trait and covariates, while returning the estimate effect of the trait and test statistics. We further accelerate simple linear regression by solving it through matrix and algebra operations, in a way that the CpG sites are tested in chunks and only relevant parameters are computed. That said, for same CpG list, `rlm_ewas_outcome()` would take longer to finish as it performs a “site by site” test. Here we illustrate an example of simple linear regression, with the other assuming the same usage.

```
ewas_lm <- lm_ewas_outcome(db_obj=mlist,trait='bmi',covariates_string =
c('age','sex'),out_position=c('CHR','MAPINFO'))
kable(head(ewas_lm))
```

CpG	estimate	se	t_stat	t_stat_df	p_value	fdr_bh	CHR	MAPINFO
cg00207921	0.0038420	0.0014895	2.5794677	96	0.0114122	0.1703492	1	1098992
cg00223952	0.0001255	0.0002200	0.5703917	96	0.5697447	0.7681950	Y	4868996
cg00377169	-0.0000176	0.0001560	-0.1130783	96	0.9102046	0.9678487	10	71905957
cg00385063	-0.0002935	0.0001972	-1.4886033	96	0.1398696	0.4047138	21	34852302

As aforementioned, by specifying the CpG position in chromosome (`MAPINFO`), the output can be easily channeld to construct visualization plots.

```
library(qqman)
library(CMplot)
qq(ewas_lm$p_value,main = "Q-Q plot of linear model", xlim = c(0, 4), ylim = c(0, 6), pch =
18, col = "blue4")
```

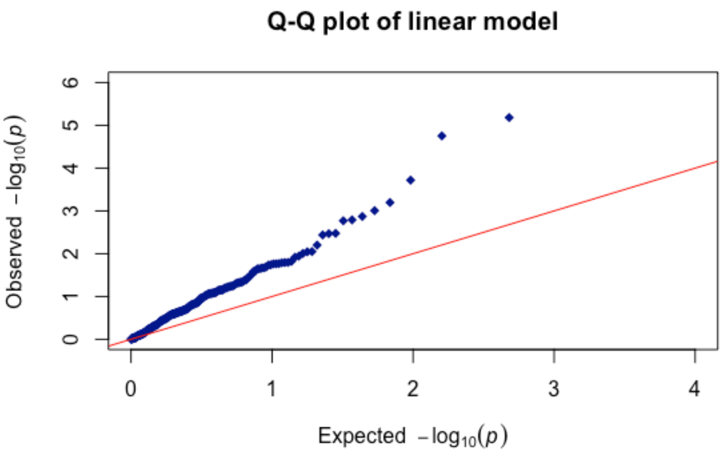


```
CMplot(ewas_lm[,c(1,8,9,6)],type='p',plot.type='m',dpi=300,file.output = FALSE,
       verbose=TRUE,width=400,height=172,chr.labels.angle=45,main = 'Manhattan_linear')
#>  Rectangular Manhattan plotting p_value.
```

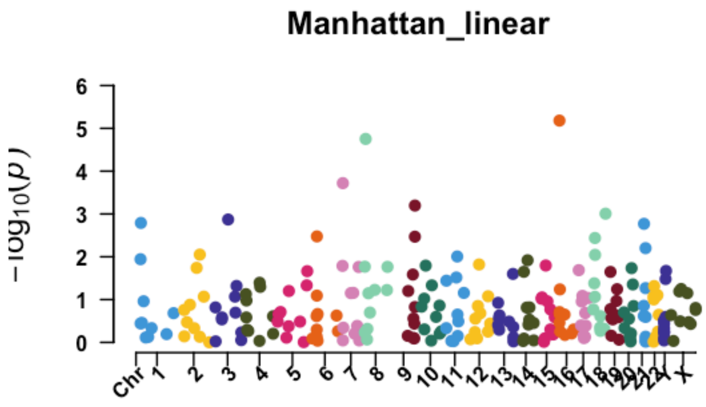

Mixed linear model

As inspired by GENESIS, there are two steps involved when fitting a mixed model to test the association:

- `NullModel()` fits the null model without the CpG variable, where phenotypic trait is treated as the outcome. In particular, random effect is specified by covariance matrices (`cov.mat`).
- `ewas_meth_exposure()` then runs the association testing by adding each CpG to the null model and returns a data frame with coefficient estimate and test statistics.

```
# Create a random kinship matrix
library(Matrix)
library(psych)
kinship <- Matrix(runif(10000,min=-1,max=1), 100)
kinship <- forceSymmetric(kinship)
diag(kinship) <- 1-0.01*runif(100,min=-1,max=1)
row.names(kinship) <- phenoData$sample_id
colnames(kinship) <- row.names(kinship)
# Make the matrix positive definite
kinship <- cor.smooth(as.matrix(kinship))
#> Warning in cor.smooth(as.matrix(kinship)): Matrix was not positive definite,
#> smoothing was done
kable(kinship[1:4,1:4])
```

	GSM2350716	GSM1504930	GSM1174899	GSM1504938
GSM2350716	1.0000000	-0.0539433	0.0103661	-0.0858376
GSM1504930	-0.0539433	1.0000000	-0.1225220	-0.1432650
GSM1174899	0.0103661	-0.1225220	1.0000000	-0.1466359
GSM1504938	-0.0858376	-0.1432650	-0.1466359	1.0000000

Now fit the null model with `age and`sex` as fixed and kinship matrix as random effect.

```r
m.null=NullModel(db_obj=mlist,trait='bmi',covariates_string = c('age','sex'),cov.mat =
kinship,method='mixed',family='gaussian')
#> Computing Variance Component Estimates...
#> Sigma^2_A     log-lik     RSS
#> [1]   60.007455   60.007455 -390.103105    1.497068
#> [1]   13.675030   79.134497 -381.358131    1.384197
#> [1]    5.825459   88.791435 -379.920783    1.301802
#> [1]    2.651337   93.373190 -379.411805    1.266464
#> [1]    1.220248   95.571419 -379.198438    1.250303
#> [1]    0.540649   96.644481 -379.100825    1.242593
#> [1]    0.2094845   97.1742085 -379.0541457    1.2388293
#> [1]    0.04601847   97.43733918 -379.03132086    1.23696993
#> [1]    0.000000   97.502903 -379.024921    1.236557
#> [1]    0.000000  116.155481 -379.024921    1.037987
#> [1]    0.000000  120.406395 -379.024921    1.001341
#> [1]    0.000000  120.567658 -379.024921    1.000002
names(m.null)
#> [1] "NullModel" "Y"         "X"
m.null$NullModel$model$formula
#> [1] "bmi ~ age + sex + (1|A)"
```

Run association tests with fitted null model on chromosome 1-5.

```r
chr <- as.character(seq(from=1,to=5))
mixed=ewas_meth_exposure(db_obj=mlist,m.null,select_sites = FALSE,select_chr = chr,
out_position=c('MAPINFO','Gene'))
kable(head(mixed))
```

CpG	Estimate	Score	Score.Stat	p_value	fdr_bh	CHR	MAPINFO	Gene
cg00207921	16.87042	0.3726769	2.507432	0.0121612	0.1520150	1	1098992	
cg01060026	-16.28013	-0.1816404	-1.719630	0.0854997	0.3057440	3	129324501	PLXND1
cg01181817	-14.08099	-0.0905021	-1.128875	0.2589504	0.4979816	3	49941376	MST1R
cg01468656	-14.41760	-0.1779558	-1.601779	0.1092045	0.3412641	1	19991678	HTR6

Users have great flexibility in customizing visualization. For instance, with gene name listed in the output, one can plot the CpG sites with gene as label using ggplot2. The CpGs on the top would be those with lowest p-values.

```r
ggplot(mixed, aes(x=CHR, y=-log10(p_value))) +
  geom_point(aes(color=as.factor(CHR))) +
  geom_text(label=mixed$Gene,size=2,vjust=2) + theme(legend.position="none")
```

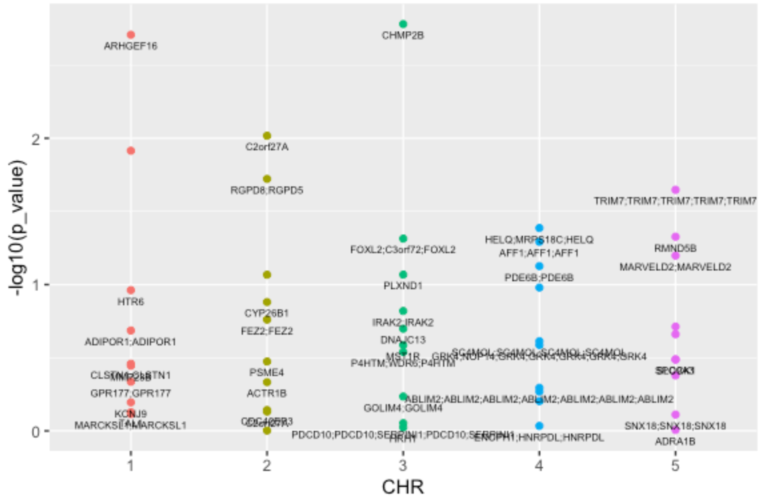

Flexible EWAS

`flex_ewas` tests the CpG-phenotype association one by one using the provided external model. For example, the following robust linear regression takes methylation as exposure to compute its association with a quantitative trait `height`.

```
rlm_function <- function(x) {
  mod <- base::suppressWarnings(MASS::rlm(as.formula(paste('height', "~x+", 'age')), data =
phenoData))
  return(summary(mod)$coefficients[2,])
}

flex_rlm <- flex_ewas(mlist,rlm_function,out_position = c('MAPINFO','Gene'))
kable(head(flex_rlm))
```

CpG	Value	Std. Error	t value	CHR	MAPINFO	Gene
cg00207921	-0.0756348	7.664024	-0.0098688	1	1098992	
cg00223952	39.2807059	37.354434	1.0515674	Y	4868996	PCDH11Y
cg00377169	110.3342063	103.281388	1.0682874	10	71905957	TYSND1;TYSND1
cg00385063	57.2468128	57.054041	1.0033788	21	34852302	TMEM50B

Using MethParquet for Methylation Risk Score and Differentially Methylated Regions

This part of the running example shows how to use MethParquet to compute methylation risk score (MRS) and analyze differentially methylated regions (DMRs) based on normalized methylation data. To detect DMRs we incorporated the software Aclust2.0.

For simplicity, this example starts after creation of MethList.

Methylation Risk score (MRS)

`dev_meth_score()` can be used to compute MRS as weighted sum of an individual's DNAm values and pre-calculated weights associated with the phenotype of interest. Then `test_mrs` can test the MRS as an exposure for its association with a chosen phenotype, whether continuous or categorical, by fitting a linear, logistic or multinomial regression model.

Compute MRS

Here we illustrate an example of computing a MRS for body mass index (BMI), and test its association with BMI. Note that for interpretation purposes, we can choose to scale the MRS.

```
data(ewas_bmi) # Load data for BMI associated CpGs

mrs_bmi <- dev_meth_score(db_obj=mlist,cpg_col='CpG',meth_effect_data = ewas_bmi,effect_col =
'Effect', NAs_to_zero = TRUE,scale_score = TRUE)
kable(head(mrs_bmi))
```

GSM1505103	0.1578201
GSM1505113	-1.2952102
GSM1505099	0.1640210
GSM1505117	-0.8833481
GSM1505102	-0.1880272
GSM1505101	-1.3378684

Test MRS with a phenotype

Results of `test_mrs` is a list containing fitted model and the coefficients for the MRS. As shown below, the MRS for BMI showed a significant association with BMI for this example dataset.

```
test_mrsBMI=test_mrs(db_obj = F,phe_data = mlist$subject_annot, outcome = 'bmi',covariates =
F,mrs = mrs_bmi)
summary(test_mrsBMI[[1]])
#>
#> Call:
#> lm(formula = y ~ x)
#>
#> Residuals:
#>     Min      1Q  Median      3Q     Max
#> -25.611  -6.842  -0.928   5.955  32.532
#>
#> Coefficients: (1 not defined because of singularities)
#>              Estimate Std. Error t value Pr(>|t|)
#> (Intercept)    40.894      1.124  36.377  < 2e-16 ***
#> x(Intercept)       NA         NA      NA       NA
#> xmrs           -4.245      1.130  -3.757 0.000292 ***
#> ---
#> Signif. codes:  0 '***' 0.001 '**' 0.01 '*' 0.05 '.' 0.1 ' ' 1
#>
#> Residual standard error: 11.24 on 98 degrees of freedom
#> Multiple R-squared:  0.1259, Adjusted R-squared:  0.117
```

```
#> F-statistic: 14.12 on 1 and 98 DF,  p-value: 0.000292
kable(test_mrsBMI[[2]])
```

	mrs
Estimate	-4.244919
Std. Error	1.129836
t value	-3.757111
Pr(>|t|)	0.000292

Differentially Methylated Regions (DMRs)

The software [Aclust2.0]() requires methylation data in the format that the rows and columns are CpGs and samples IDs. Since it's a rather time consuming process for DMR analysis, we provided a small toy data for DMR analysis. Here we provide an example code to obtain the betas data using `cpg_extract`.

```
# Example code to generate subset of methylation data

# Sampling 5000 CpGs from CpG Annotation file
# cl<-mlist$cpg_annot %>% group_by('CHR') %>% sample_n(5000)

# Extract these 5000 CpGs
# extr<-cpg_extract(mlist,site_names=cl$Name)

# Get methylation data
# betas<-extr$cpg_data %>% dplyr::select(-CHR) %>% remove_rownames() %>%
column_to_rownames('CpG')

# Resulted data:
betas_DMR<-read.csv(paste0(wdir,'/inst/extdata/MethData_DMR.csv'),row.names=1)
kable(head(betas_DMR[,1:3]))
```

	GSM1505103	GSM1505113	GSM1505099
cg14657517	0.034	0.062	0.043
cg09810313	0.021	0.021	0.011
cg27186774	0.007	0.017	0.012
cg05592035	0.416	0.428	0.477

The following script are from the `aclust2.0.R` script of the Aclust2.0 software.

Step 1:Call functions & import betas —————————————-

Download the code from Github into a named directory and set as working directory.

```
library(tidyverse)
library(readr)
require(geepack)
require(data.table)
library("biomaRt")
library("ChIPpeakAnno")
library("GenomicRanges")
library(tibble)
require('knitr')
setwd(path_to_Aclust2.0-main)
source(paste0(path_to_Aclust2.0-main,'aclust2.0_utils.R'))
```

Step 2: Get Illumina manifests for MM285, 450K, EPICv1, EPICv2 ———

```
manifest <- get_manifest("EPICv1") #to pull EPICv1 manifest
```

Step 3: Clustering of CpGs ——————————————————-

```
##This step creates a list that accommodates three objects
list.out <- find_cluster_list(probe.vec = rownames(betas_DMR),
                              betas = betas_DMR,
                              manifest = manifest,
                              minimum.cluster.size = 2)
clusters.list <- list.out$clusters.list #required for optional GEE model in step 4
annot.betas <- list.out$annot.betas #required chromosomal annotation in step 5
cpg.clusters <- list.out$cpg.clusters #Includes only the CpG clusters which can be passed
directly as clus object for gene annotations (step 5)
```

Step 4: Run gee model ——————————————————————————

```
## Define exposure, covariates (optional) and sample.id
pheno<-read.csv(paste0(wdir,'/inst/extdata/PhenoData_DMR.csv'),row.names=1)
sample.id  <- pheno$sample_id
exposure <- pheno$bmi #(can also take categorical exposure variables)
covariates<- pheno %>% dplyr::select(age, sex)
identical(colnames(betas_DMR), sample.id) ##must be in the same order

###Adjusting for covariates
cluster.gee <- GEE.clusters(betas = betas_DMR, clusters.list = list.out$clusters.list,
                            exposure = exposure,covariates= covariates,
                            id = colnames(betas_DMR), working.cor = "ex",
                            sample.id = sample.id) %>%
  mutate(exposure_padjusted = p.adjust(exposure_pvalue, "BH")) ##adjusting for false positives
```

Step 5: Perform chromosomal annotations ————————————————

```
#For cluster outputs from gee model in step 4
annot.betas <- list.out$annot.betas
clus <- cluster.gee ## (from step 4)
annotated.genes <- annot.clus.gene(annot.betas = annot.betas,
                                   clus = clus, model = "hsa")
```

Obtained DMR results by genes:

```
annotated.genes <-read.csv(paste0(wdir,'/inst/extdata/DMR_results.csv'))
kable(annotated.genes)
```

feature	external_gene_name	seqnames	start	end	width	strand	probe	cluster_name
ENSE00001174442	IL6R	chr1	154404902	154404926	25	*	2	cluster_1
ENSE00001338726	IRX6	chr16	55328429	55328841	413	*	2	cluster_5
ENSE00001403134	ATP2A1	chr16	28878308	28878778	471	*	2	cluster_4
ENSE00001425120	PABPC3	chr13	25096044	25096048	5	*	2	cluster_3
ENSE00001560063	FTSJ1	chrX	48475919	48476024	106	*	2	cluster_8

feature_strand	insideFeature	distancetoFeature	shortestDistance	fromOverlappingOrNearest
+	upstream	-279	267	NearestLocation
+	overlapStart	-65	141	NearestLocation
+	overlapStart	55	11	NearestLocation
+	upstream	-90	88	NearestLocation
+	overlapStart	-49	3	NearestLocation
